# Staged treatment and internal fixation of floating ankle: A case report and literature review

**DOI:** 10.1097/MD.0000000000023704

**Published:** 2020-12-11

**Authors:** Jihang Yao, Tao Jin, Chengfu Song, Weina Ju, Zhisen Tian, Baochang Qi, Yuanyi Wang

**Affiliations:** a Department of Orthopedic Traumatology; b Department of Neurology, the First Hospital of Jilin University; c Department of Orthopedics, Dehui Traditional Chinese Medicine Hospital; d Department of Spine Surgery, China-Japan Union Hospital of Jilin University; e Department of Spine Surgery, the First Hospital of Jilin University; f Jilin Engineering Research Center For Spine And Spinal Cord Injury.

**Keywords:** distal tibial fracture, Floating ankle, multiple metatarsal fracture, open fracture, staged treatment

## Abstract

**Rationale:**

Floating ankle is a rare traumatic condition characterized by a combination of tibial and ipsilateral foot fractures, with the ankle remaining intact. It is usually caused by high-energy trauma and also presents with serious soft tissue damage. Its treatment is mainly restricted to external fixation, which results in poor outcomes. We present a patient with a floating ankle who underwent staged treatment and achieved full internal fixation, subsequently returning to normal activity.

**Patient concerns:**

A 26 year- old man had an accident with an reel machine and sustained an open fracture on his right lower extremity.

**Diagnoses:**

Digital radiograph demonstrated a distal tibial fracture, fibular fracture, and multiple metatarsal fractures, which fulfilled the criteria for a floating ankle.

**Interventions:**

Initial ankle-spanning external fixation was performed. After 21 days, the patient underwent open reduction and internal fixation on his first and fifth metatarsals, and K-wire fixation on his fourth metatarsal. The external fixator was replaced by plaster fixation. Seven days later, the patient underwent internal fixation of his leg, open reduction and internal fixation with plating was applied of the fibular fracture, and minimally invasive plate osteosynthesis of the tibial fracture.

**Outcomes:**

At 1-year follow-up, bone union was identified by digital radiograph; after 2 years, his ankle function had fully recovered, and he resumed his normal activities.

**Lessons:**

In the staged treatment protocol of the floating ankle, temporary external fixation provided traction and immobilization of the skeletal and soft tissues. Secondary internal fixation maintained the reduction and alignment and allowed early exercise, which is critical to the prognosis of a floating ankle.

## 1 Introduction

Floating ankle is an extremely rare traumatic condition, defined as a distal tibial fracture and an ipsilateral foot fracture with an intact ankle. This under-recognized trauma was first described by McHale et al. in 2002 as a war-related injury.^[1]^ The fracture combination renders the ankle a separate anatomical entity with instability. As the floating ankle is caused by a high-energy accident, it is often combined with soft tissue damage, 1 of the main obstacles of bone healing and functional recovery. In previous studies, clinicians successfully converted the treatment of floating ankle with a severe open fracture from amputation to limb salvage; however, poor outcomes and complications were reported.^[2]^ With the advancement of traumatic surgery, we can now manage injuries in staged protocols, in which appropriate procedures are applied in different stages using suitable techniques.

We present a case of floating ankle with an open distal tibial, fibular, and multiple metatarsal fractures. To the best of our knowledge, the twisting mechanism of the injury has not been previously described. In the treatment protocol, a series of 4 fixation methods was performed step by step, and full internal fixation was achieved, allowing earlier postoperative exercise, and leading to favorable outcome in follow-ups.

## 2 Case report

**Figure 1:**
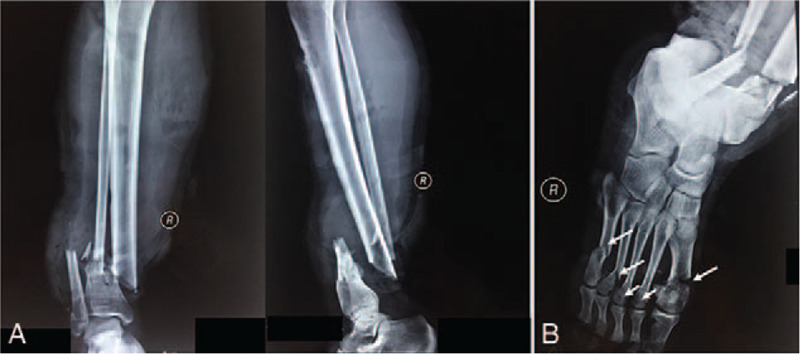
Digital radiograph of the patient's right lower extremity, demonstrating open tibial, fibular, and metatarsal fractures with an intact ankle complex, which conforms to the definition of a floating ankle. The anterior and lateral planes of the tibia and fibula demonstrated an AO 42b3 fracture with anterior dislocation (A). Digital radiograph of the right foot showing fracture of all metatarsals (B, arrows). The ankle mortise view remained intact (A and B).

A 26-year-old man with a traumatic history requested a consultation at our hospital. The patient was injured by a reel machine and sustained an open fracture on his right lower extremity. The patient's right leg presented with extensive open injury and edema. He was unable to move his right ankle and had limited movement of the metatarsal joints. The digital radiograph demonstrated fractures of the right tibia, fibula, and multiple metatarsals (Fig. 1). The patient was diagnosed with a floating ankle with a Gustilo grade II open fracture, right tibial, fibular, and multiple metatarsal fractures.

**Figure 2:**
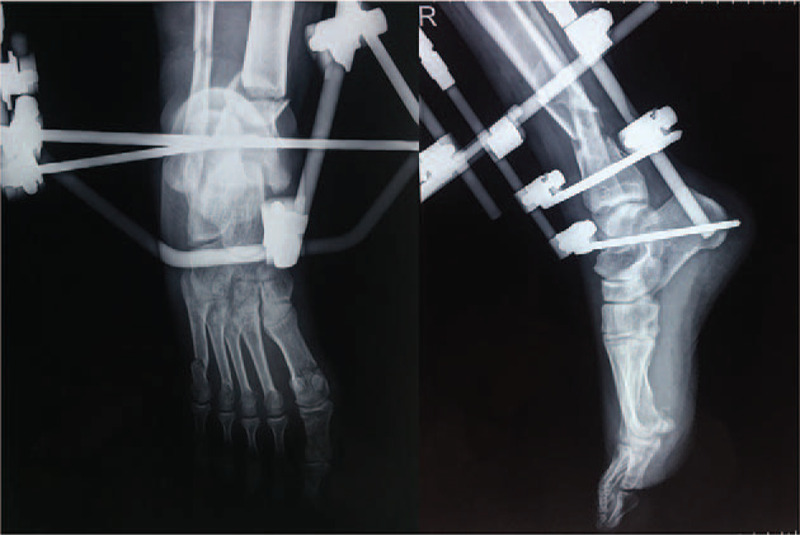
Digital radiograph at day 14 post-injury. Dislocations of the tibia and fibula were partially corrected under traction using an ankle-spanning external fixator.

**Figure 3:**
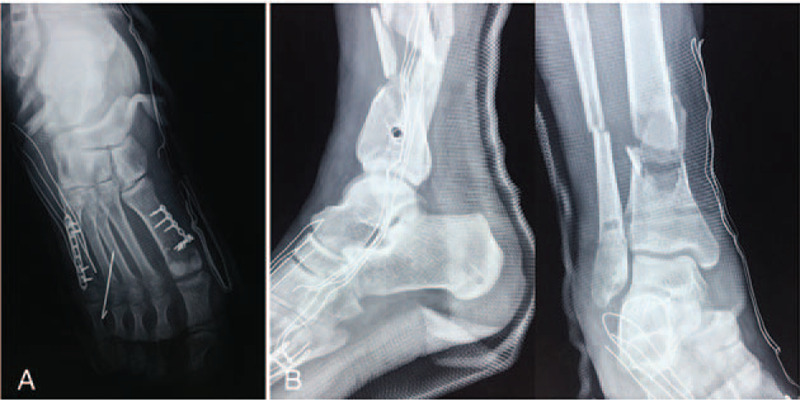
Digital radiograph of the patient's right foot after open reduction internal fixation (ORIF) and external fixator removal. ORIF was applied to the first and fifth metatarsals, and the fourth metatarsal was fixed by percutaneous K-wire insertion (A). The remaining 2 fractured metatarsals, tibia, and fibula were fixed by plaster instead of an external fixator (B).

**Figure 4:**
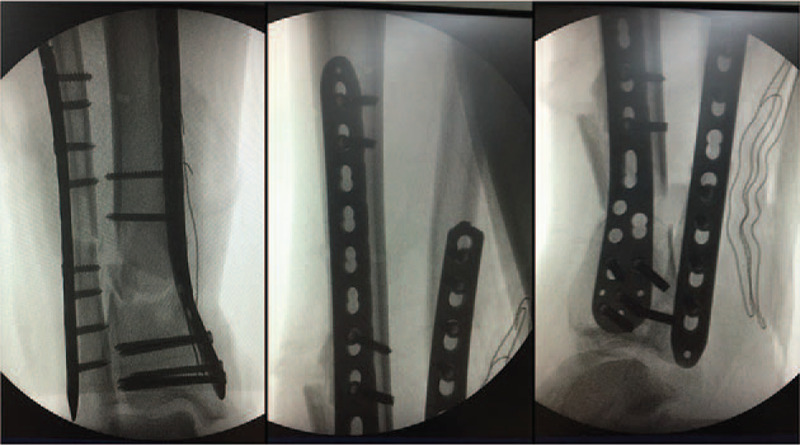
Intraoperative fluoroscopy during surgery of the fibular and tibial fractures. Open reduction internal fixation and minimally invasive plate osteosynthesis techniques were performed, with reduction and internal fixation of the fibula and tibia, and the alignment was corrected via open and closed reduction, respectively.

Considering the great distance to our center, he underwent debridement and ankle-spanning external fixation at a local clinic. After 14 days, the patient was transferred to our department for further intervention, with his soft tissue injuries and tibial fracture stable under the traction of the external fixator (Fig. 2). On day 21 post-injury, he underwent an open reduction and internal fixation (ORIF) on his first and fifth metatarsals and percutaneous K-wire fixation on his fourth metatarsal (Fig. 3A). After foot surgery, the external fixator was removed and replaced by plaster fixation (Fig. 3B). On day 28 post-injury, the patient underwent internal fixation, minimally invasive plate osteosynthesis (MIPO) of the tibial fracture, and open reduction with plating of the fibular fracture (Fig. 4).

**Figure 5:**
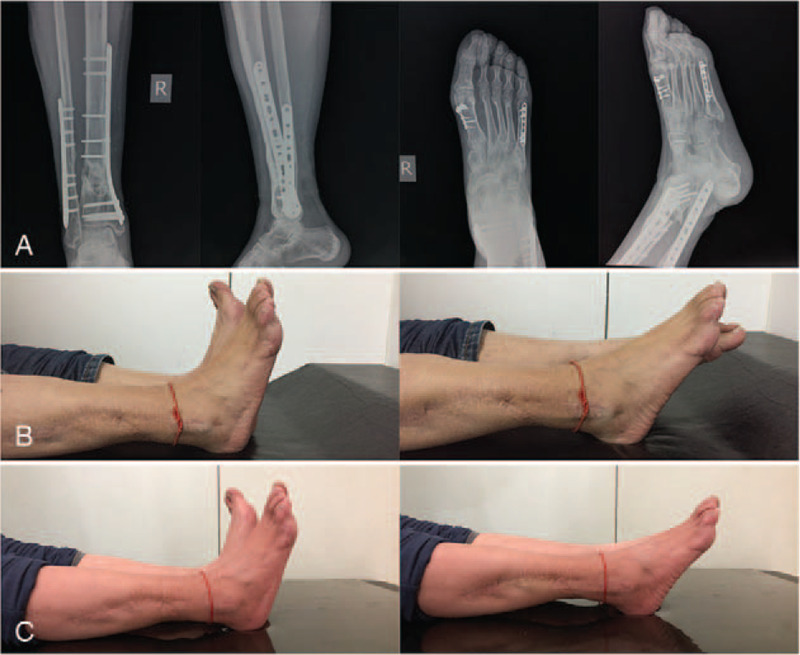
Digital radiograph and motion pictures at 1-year follow-up. Digital radiograph of the lower extremity demonstrates bone union and corrected alignment at the fracture sites on the tibia, fibula, and metatarsals (A). Functional improvement was significant over the first year; however, the flexion and extension of the ankle remained restricted when compared with the contralateral limb (B). At the 2-year follow-up, the function of his right lower extremity was basically essentially fully recovered (C).

The patient started non-weight-bearing and passive ankle movement exercises 2 weeks postoperatively. After 3 months, the ankle movement improved significantly, the K-wire was removed, and he started weight-bearing exercises as tolerated. At 1-year follow-up, the digital radiograph demonstrated bone union and correct alignment (Fig. 5A). His ankle motion gradually recovered over the first year (Fig. 5B), and at the 2-year follow-up, the motion was almost identical to that of the contralateral ankle, and the patient was able to fully resume his normal activities (Fig. 5C).

## 3 Discussion

As a rare traumatic injury of the lower extremity, floating ankle is inadequately documented. Including the current case, only 13 cases (11 men and 2 women) have been reported since 2002 (Table 1). Twelve of these patients were 18–40 years old, and 9 were military personnel or those who undertook high-risk activities (operators of heavy machinery or motorcycle riders). The floating ankle had similar mechanisms of injury to other “floating” patterns resulting from high-energy violence, among which motor accidents and fall from a height are typical causes. In the literature, all patients with floating ankles sustained injuries from direct high-energy trauma, including explosion and blast injuries, falls, and vehicular accidents (Table 1). The present case has a unique mechanism of injury, in which the lever effect of the reel led to anterior dislocation of the distal tibia and fracture of 5 metatarsals. Based on our extensive review, such a mechanism has not been mentioned in the literature.

**Table 1:** Review of “floating ankle” injury cases.

Citation	Age & gender	Mechanism	Leg injury	Foot injury	Treatment	Postoperative course
1	24 M	N/A	Comminuted grade I fracture of the distal tibia	Amputating of the fifth MT and fractures at remaining MTs	External fixation;	Underwent foot stabilizing surgery after 1 yr and amputated due to infection
1	37 M	Explosion	Grade IIIB fracture of the distal tibia and fibula	Ipsilateral comminuted fracture of the calcaneus	Leg external fixation; calcaneus internal fixation	Returned to full duty
1	21 M	Explosion	Fracture of distal tibia and fibula	Open, comminuted calcaneus and talus fractures	IMN treatment failure, external fixation around IMN	The patient had painful exostoses from the calcaneal fracture.
1	9 F	Truck accident	Fracture of the distal tibia	Open calcaneus fracture with significant soft tissue loss at the heel	External fixation	Excellent outcome. The patient returned to all normal activities.
2	18 M	Motorbike accident	Grade III open fractures of the distal tibia	Multiple MT fracture	Ilizarov and ORIF fixation	Bone union after 4 mo with superficial pin site infection
2	23 F	Road traffic accident	Grade III open fractures of the distal tibia	Calcaneal fracture	Hoffman and ORIF fixation	Bone union after 4 mo with painful ankle and heel
2	25 M	Motorbike accident	Grade III open fractures of the distal tibia	Multiple MT fractures	Hoffman and ORIF fixation	Bone union after 4 mo without complications
2	28 M	Motorbike accident	Grade III open fractures of the distal tibia	Multiple MT fractures	Hoffman and ORIF fixation	Bone union after 5 mo with superficial pin site infection
2	29 M	Motorbike accident	Grade III open fractures of the distal tibia	Multiple MT fractures	Hoffman fixation	Bone union after 4 mo without complications
2	32 M	Fall from height	Grade III open fractures of the distal tibia	Lisfranc fracture; multiple MT fractures	Ilizarov and ORIF fixation	Bone union after 6 mo with superficial pin site infection
2	34 M	Motorbike accident	Grade III open fractures of the distal tibia	Multiple MT fractures	Hybrid and Mini ORIF fixation	Delayed bone union at 9 mo with 1st MT osteomyelitis
2	35 M	Road traffic accident	Grade III open fractures of the distal tibia	Calcaneal fracture	Ilizarov and ORIF fixation	Bone union after 6 mo with painful ankle and heel
Present case	26 M	Twisting violence of machine	Grade II open Right distal tibia and fibula comminuted fracture	Multiple MT fractures	Initial external fixation, ORIF for fibula fracture, ORIF and K-wire fixation for metatarsals fractures, MIPO for tibial fixation	Bone union after 1 yr, return to normal activities

CRIF = closed reduction and internal fixation, CRPS = complex regional pain syndrome, F = female, M = male, MT = metatarsal, ORIF = open reduction and internal fixation. 1. The “Floating Ankle”: A Pattern of Violent Injury. Treatment with Thin-Pin External Fixation. 2. Open grade III “floating ankle” injuries: a report of eight cases with review of literature.

According to the definition of the floating ankle, distal tibial fracture is an indispensable requirement, which presents as simple to comminuted fractures with/without fibular fractures (Table 1). A distal tibial fracture in a floating ankle is difficult to treat due to the injury's complexity, lack of muscle coverage, poor vascularity, and disruption of foot fractures.^[3]^ In the literature, all patients had open fractures, among which 9 had a Gustilo type III open fracture (Table 1), suggesting poorer soft tissue coverage of the fracture; and 3 patients had not only a tibial but also fibular fractures, indicating additional instability. Hence, appropriate treatment strategies are important for the prognosis of patients with floating ankles. Devitalization of the soft tissue envelope is often seen in floating ankle cases; therefore, surgical management usually starts with irrigation, debridement, and external fixation.^[3]^ Ankle-spanning external fixators improve soft tissue healing, prevent soft tissue compromise, and stabilize the bone alignment without making incisions around the site of trauma. Initial external fixation should be performed in local facilities before transfer, which was ensured in this case by using our hospital's distance consultation system. However, McHale et al reported that inadequate immobilization and poorly done debridement greatly hindered continual treatment and caused poor outcomes, emphasizing the importance of primary interventions by experienced specialists.^[1]^

Although external fixation is a useful temporary option for skeletal and soft tissue traction, it provides less stability than does internal fixation.^[4]^ Moreover, external fixation may cause unfavorable outcomes, including mal- and non-union, pinpoint infection, and ankle stiffness.^[5]^ In previous floating ankle cases treated with external fixation, only 4 patients resumed normal activities in follow-ups, which suggested that a more definitive fixation method was necessary. Over 90% of patients with distal tibial fractures underwent internal fixation as a secondary procedure to replace primary external fixation when evidence of soft tissue recovery emerged. In a floating ankle, we cannot directly perform the replacement, because the instability caused by foot fractures makes tibial immobilization difficult, or vice versa.^[1]^ External fixation is an ideal solution to the double-end instability dilemma. Under the immobilization provided by the external fixator, the foot fracture can be reduced without disturbing the tibial instability; likewise, this eliminates the instability from the ipsilateral foot, while applying internal fixation to the fractured tibia. Consequently, the foot fracture should be reduced after preliminary fixation and prior to definitive fixation of the tibia.

As an essential component of floating ankle, the foot fracture dissociates the distal end of the floating anatomical entity. In published reports, the foot fracture altered the calcaneal, talar, and metatarsal fractures, among which metatarsal fracture is the predominant foot fracture pattern in the floating ankle (Table 1). Metatarsal fracture is a common injury, with favorable prognosis if properly assessed.^[6]^ Due to the splinting effect of the adjacent metatarsals, referral is not necessary for a simple fracture of a single metatarsal, unless the translocation is more than 3–4 mm, or the dorsal angulation is >10°.^[7,8]^ However, fractures on the first and fifth metatarsals require more careful treatment due to their unique structure and functions. The first metatarsal bone carries twice the load of the other 4 during walking;^[6]^ therefore, dislocation and instability in the first metatarsal are less well tolerated than in other metatarsals. Because of the traction of the flexor tendons and intrinsic muscles, the distal fragment is often displaced in the plantar direction and is difficult to realign; consequently, internal fixation with plating is necessary. Internal fixation of the fifth metatarsal fracture is also essential, due to the wide range of motion and loose ligamentous connection. In our case, all the metatarsals were fractured following the twisting mechanism of the accident; the first, fourth, and fifth metatarsals on the shaft; and the second and third metatarsals on the heads without displacement. Therefore, different fixation methods, including ORIF with plating, closed reduction with K-wire fixation, and plaster immobilization were employed in the metatarsals, which provided reliable stability for the subsequent tibial surgery.

The reduction and fixation of the foot fracture offered good timing for further internal fixation of the tibia and fibula. The common internal fixation technique of the distal tibial fracture includes intramedullary nailing (IMN), ORIF, and MIPO. Extensive incision in ORIF may disrupt the surrounding soft tissue, extraosseous vessels, and fracture fragments, which contribute to the development of several complications, including non-union, malunion, and infection.^[9]^ IMN is more suitable for treating diaphyseal fractures than metaphyseal fractures. Nevertheless, a longer lever arm, difficult realignment, metaphyseal enlargement, high rate of knee complications, and high risk of reduction defects excludes IMN as an ideal intervention for distal tibial fractures.^[10]^ Compared with the other 2 options for internal fixation, MIPO offers reliable reduction via minimal incisions relatively distant to the injury site, avoids damage induced by the anterior incision of ORIF, and retains the biological environment of the fracture healing.^[11]^ MIPO is also not associated with the complications of IMN, including knee complications, metaphyseal enlargement, and malalignment. MIPO was reported to have satisfactory outcomes in the distal tibial fracture, including a high union rate, and good functional recovery with fewer complications.^[12–14]^ In the current case, the patient had a grade II open distal tibial fracture, which completely fulfilled the indications of MIPO. However, MIPO is not optimal in cases of floating ankle with soft tissue defects or severely comminuted distal tibia fractures due to the high risk of reduction failure and occurrence of soft tissue complications.^[9]^ This may explain why external fixations were preferred in earlier floating ankle cases (Table 1).

Our patient underwent full internal fixation of his fractures, which ensured the maintenance of the reduction. Additionally, as per the different conditions and principles of the fractures, the patient underwent 4 fixation methods in the treatment protocol, including initial external fixation, ORIF for the fractures of the fibula and metatarsals, MIPO for the distal tibial fracture, and plaster fixation for the 2 undisplaced metatarsal fractures. In the treatment of the floating ankle, 2 concepts were extracted from our experience:

(1) in the whole-separate concept, different injuries should be treated in the most appropriate method when the general fracture condition is stabilized; and
(2) the leg-foot-leg concept means that the treatment of the floating ankle starts with the ankle-spanning external fixation, is relayed by foot reduction and fixation, and ends with internal fixation of the leg.

The main advantage of our protocol is the allowance of early passive and active ankle exercises, which not only prevented complications including stiffness caused by long-time external fixation,^[1]^ but also maintained the ankle's motion range and flexibility. The major limitation of the study is the low number of reported cases and published literature on the floating ankle. The limited information on mechanisms of injury and treatment options prevent impede our ability to draw further conclusions.

## 4 Conclusions

As a rare multiple injury in lower extremities with a distinct anatomical entity, cases of floating ankle are inadequately documented, especially in the last 2 decades. We report a case of floating ankle injured by an unreported mechanism of twisting trauma. We applied different fixation to the various fractures in the appropriate stages, maintained the reduction using full internal fixation, and facilitated early postoperative exercise, ultimately causing favorable outcomes.

## Author contributions

**Conceptualization:** Baochang Qi, Yuanyi Wang.

**Data curation:** Weina Ju.

**Methodology:** Baochang Qi.

**Project administration:** Yuanyi Wang.

**Resources:** Chengfu Song.

**Software:** Weina Ju, Zhisen Tian.

**Supervision:** Yuanyi Wang.

**Visualization:** Zhisen Tian.

**Writing – original draft:** Jihang Yao, Tao Jin.

**Writing – review & editing:** Yuanyi Wang.
